# Contact of Teeth With Blades of Macintosh Laryngoscope, McGrath MAC® Video Laryngoscope, and Pentax-AWS Airway Scope® During Tracheal Intubation: A Randomized Crossover Simulation Study

**DOI:** 10.7759/cureus.91688

**Published:** 2025-09-05

**Authors:** Noriya Hirose, Miki Matsui, Tomoaki Itaya, Keisuke Nakazawa, Shunichi Takagi, Takahiro Suzuki

**Affiliations:** 1 Anesthesiology, Nihon University School of Medicine, Tokyo, JPN

**Keywords:** dental injury, general anesthesia, laryngoscopy, tracheal intubation, video laryngoscope

## Abstract

Background

Dental injury is a common complication during laryngoscopy for tracheal intubation. Due to the variety of laryngoscopes, the site of tooth contact with the laryngoscope blade might vary. The aim of this study was to investigate the difference in contact status of each individual tooth and various laryngoscope blades during orotracheal intubation, evaluated using a simulation study.

Methods

Fifteen anesthesiologists randomly attempted orotracheal intubation in two types of simulators (models A and B) using a Macintosh laryngoscope with #3 blade (ML) (Riester, Jungingen, Germany), McGrath MAC® video laryngoscope with #3 blade (MAC) (Medtronic, Covidien, Tokyo, Japan), and Pentax-AWS Airway Scope® with SL blade (AWS) (Nihon Koden, Tokyo, Japan), with colored articulating papers attached to the front and back of their blades. Following the procedure, the teeth of the simulators were examined for color transference, and the findings were recorded.

Result

The mean number (standard deviation) of contacted teeth per intubation maneuver when using the ML, MAC, and AWS were 4.3 (1.8), 3.9 (1.5), and 12.9 (2.7) in model A and 4.2 (2.3), 3.7 (1.3), and 13.2 (2.5) in model B. In both models, higher contact rates were seen for the left maxillary central incisor (>80%) and left mandibular central incisor (>60%), with no contact with the molars when using the ML and MAC. Use of the AWS resulted in higher contact rates with the central and lateral incisors bilaterally, both maxillary and mandibular (>80%), with bilateral maxillary premolars sometimes contacting the blade (>20%).

Conclusion

This simulation study suggested that during tracheal intubation using ML, MAC, and AWS, both the mandibular incisors and maxillary incisors may frequently come into contact with the laryngoscope blade. In addition, when using the AWS, more teeth come into contact with the blade compared to other laryngoscopes, and there is also the possibility of contact with the molars.

## Introduction

Dental injury is a common complication during laryngoscopy for tracheal intubation, with reported incidence rates ranging from as low as approximately 0.02% to as high as about 25% [1-5]. It is well known that dental injuries are most often caused by contact between the laryngoscope blade and the teeth, with the maxillary incisors being particularly susceptible to damage [4-6]. Therefore, previous studies have investigated the contact status, including incidence and contact force, between maxillary incisors and various laryngoscope blades [7-9]. In contrast, teeth other than the maxillary incisors are occasionally damaged during tracheal intubation in clinical settings [5,6]. This fact suggests that the laryngoscope blade comes into contact with teeth other than the maxillary incisors during endotracheal intubation. Additionally, since there are many types of laryngoscopes in clinical use, the site of dental contact with the laryngoscope blade might vary based on the type of laryngoscope used. However, there are no reports of the evaluation of the contact status of the entire dental arch when using various laryngoscopes.

The aim of this study is to examine, in a simulated study, the presence or absence of contact with the teeth and the types of teeth involved during orotracheal intubation using different laryngoscopes, including the Macintosh laryngoscope (ML) (Riester, Jungingen, Germany), McGrath MAC® videolaryngoscope (MAC) (Medtronic, Covidien, Tokyo, Japan), and Pentax-AWS Airway Scope® S200NK (AWS) (Nihon Koden, Tokyo, Japan), with the aid of dental articulating paper. The primary objective was to compare the number of teeth contacted during orotracheal intubation among the three laryngoscopes. The secondary objective was to identify which teeth are most frequently contacted for each laryngoscope. Identification of the teeth that come in contact with individual laryngoscopes would be helpful for minimizing dental injury during tracheal intubation when using these laryngoscopes in clinical practice.

## Materials and methods

This randomized crossover simulation study was conducted at Nihon University Itabashi Hospital in Tokyo, Japan, by 15 anesthesiologists with experience in administering general anesthesia to more than 300 patients, as testers, and two dentists as evaluators. All participants in the experiment were provided with an experiment protocol and agreed to participate in the study after understanding the concept of the study. Additionally, all testers practiced orotracheal intubation for simulators with each type of laryngoscope used in this study once before the actual test.

**Figure 1:**
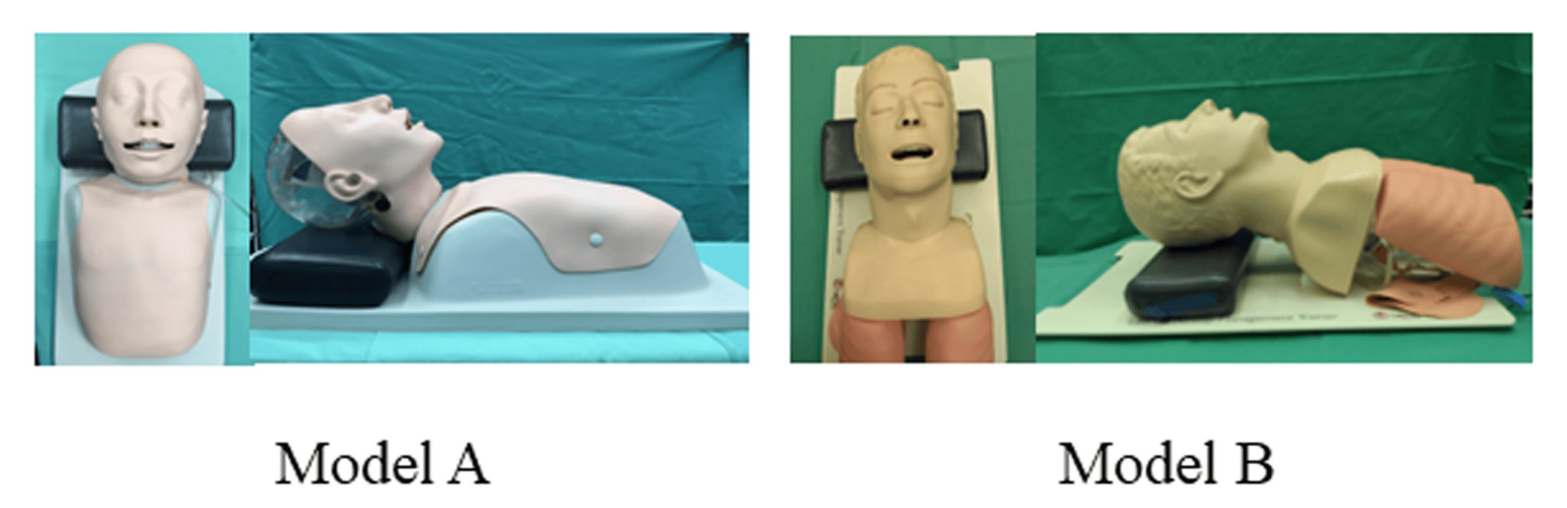
Photographs of the simulators in this study Model A: DAM simulator training model (Kyoto Kagaku, Kyoto, Japan). Model B: Airway Management Trainer (Laerdal® Medical, Stavanger, Norway).

**Figure 2:**
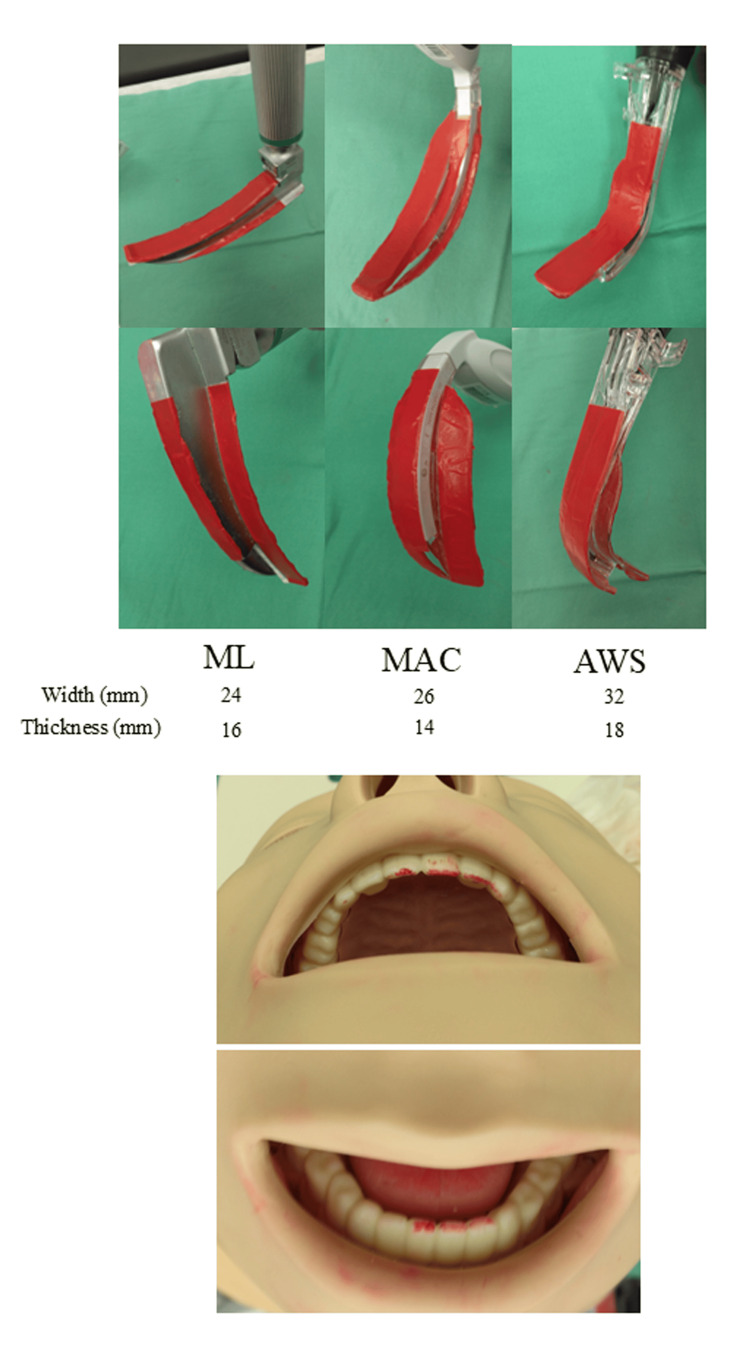
Photographs of each laryngoscope blade with articulating paper attached and colored teeth through contact with the blade Each laryngoscope blade with the attached colored articulating paper (upper photographs) and the teeth colored after contact with the blade (lower photographs; classic example).

All 15 testers attempted orotracheal intubation once each on both kinds of simulators (Figure 1), with each of the three laryngoscopes evaluated, namely, ML, MAC, and AWS, resulting in a total of six attempts per tester. The intubation attempts were performed in random order using the envelope method. Each laryngoscope was equipped with a regular-sized blade: #3 for ML, #3 for MAC, and Intlock (SL) for AWS. Additionally, colored articulating papers (GC, Tokyo, Japan), in which color transfer occurs upon contact, were attached to the front and back surfaces of the respective blades in advance (Figure 2).

Each simulation model was placed on the operation table, and a 7 cm high pillow was positioned under the head, simulating the sniffing position. After adjusting the height of the operation table, the tester performed orotracheal intubation in the presence of one of the evaluators. Mouth opening for the insertion of each laryngoscope blade was performed using the cross-finger method, and laryngoscopy was performed as in usual clinical practice. All intubations were performed using a similar-sized cuffed endotracheal tube, with an inner diameter of 7.5 mm and an outer diameter of 10.2 mm (Covidien, Tokyo, Japan). A stylet with an outer diameter of 3 mm (Fuji Systems, Tokyo, Japan) that was freely bent according to the individual testers was used for intubation when using the ML and MAC, and the Intlock was fitted with the tracheal tube beforehand when using the AWS. In each trial, the intubation maneuver was considered completed when the laryngoscope was removed from the mouth of the simulator after intubation. Subsequently, the evaluator who was present at the trial checked for colored teeth and recorded the results on a dental formula chart. In addition, if any staining was observed on the teeth, it was determined that there had been contact with the laryngoscope. The collected records were analyzed by the second evaluator, who was blinded to the laryngoscope-simulator combination used in each trial, based on the prospective randomized open blinded end-point (PROBE) method. After every trial, the colored teeth were cleaned using alcohol swabs in preparation for the next trial.

The items evaluated in this study were the total number of teeth that came in contact with the laryngoscope blade in each trial and the identification of the contacted teeth. In each of the 15 trials for each laryngoscope and simulator combination, the total number of contacted teeth was expressed as the mean (SD; standard deviation), and the frequency of contact of each tooth was expressed as the incidence (%). Differences in the total number of contacted teeth between the three laryngoscopes and between the two simulator models were analyzed using the Tukey-Kramer honestly significant difference test or the Steel-Dwass multiple comparison test and paired t-test or Wilcoxon signed-rank test, respectively. All analyses were performed using JMP version 9 (SAS, Cary, North Carolina, United States), and p-values of <0.05 were considered significant. Since there are no similar previous reports, the sample size in terms of the required number of testers was calculated based on our preliminary study using model A. In that study, the mean number (SD) of contacted teeth per trial when using the ML, MAC, and AWS were 7.5 (1.7), 7.3 (2.2), and 10.1 (2.1), respectively. Therefore, we estimated that there were no significant differences in the number of contacted teeth between using the ML and MAC. Evaluation indicated that 15 testers needed to be included in every trial using each laryngoscope, to detect the difference in the number of contacted teeth between using the ML or MAC versus the AWS, using an a priori Student t-test with a=0.016 (Bonferroni correction) and a power of 0.80.

## Results

All tracheal intubations were successfully performed, and data of dental contact status were appropriately obtained for all trials, with no damage to any of the teeth such as fractures, dislodgement, and mobility in any of the trials. The total number of contacted teeth per trial using each laryngoscope and for each simulator model is shown in Table 1. In both models, the number of contacted teeth was significantly higher when using the AWS than when using the ML (model A: p<0.0001 (95% CI: 6.8-10.5); model B: p<0.0001 (95% CI: 7.1-10.9)) and MAC (model A: p<0.0001 (95% CI: 7.2-10.9); model B: p<0.0001 (95% CI: 7.7-11.4)), although there were no significant differences when using the ML versus the MAC. Between-model comparisons indicated no significant differences in the number of contacted teeth when using each of the laryngoscopes.

**Table 1:** Total number of contacted teeth per trial Data are presented as the mean (standard deviation). ML: Macintosh laryngoscope; MAC: McGrath MAC® video laryngoscope, AWS: Pentax-AWS Airway Scope® ^*^p<0.0001 vs. ML; ^†^p<0.0001 vs. MAC (Tukey-Kramer honestly significant difference test)

	ML	MAC	AWS
Model A	4.3 (1.8)	3.9 (1.5)	12.9 (2.7)^*,†^
Model B	4.2 (2.3)	3.7 (1.3)	13.2 (2.5)^*,†^

**Figure 3:**
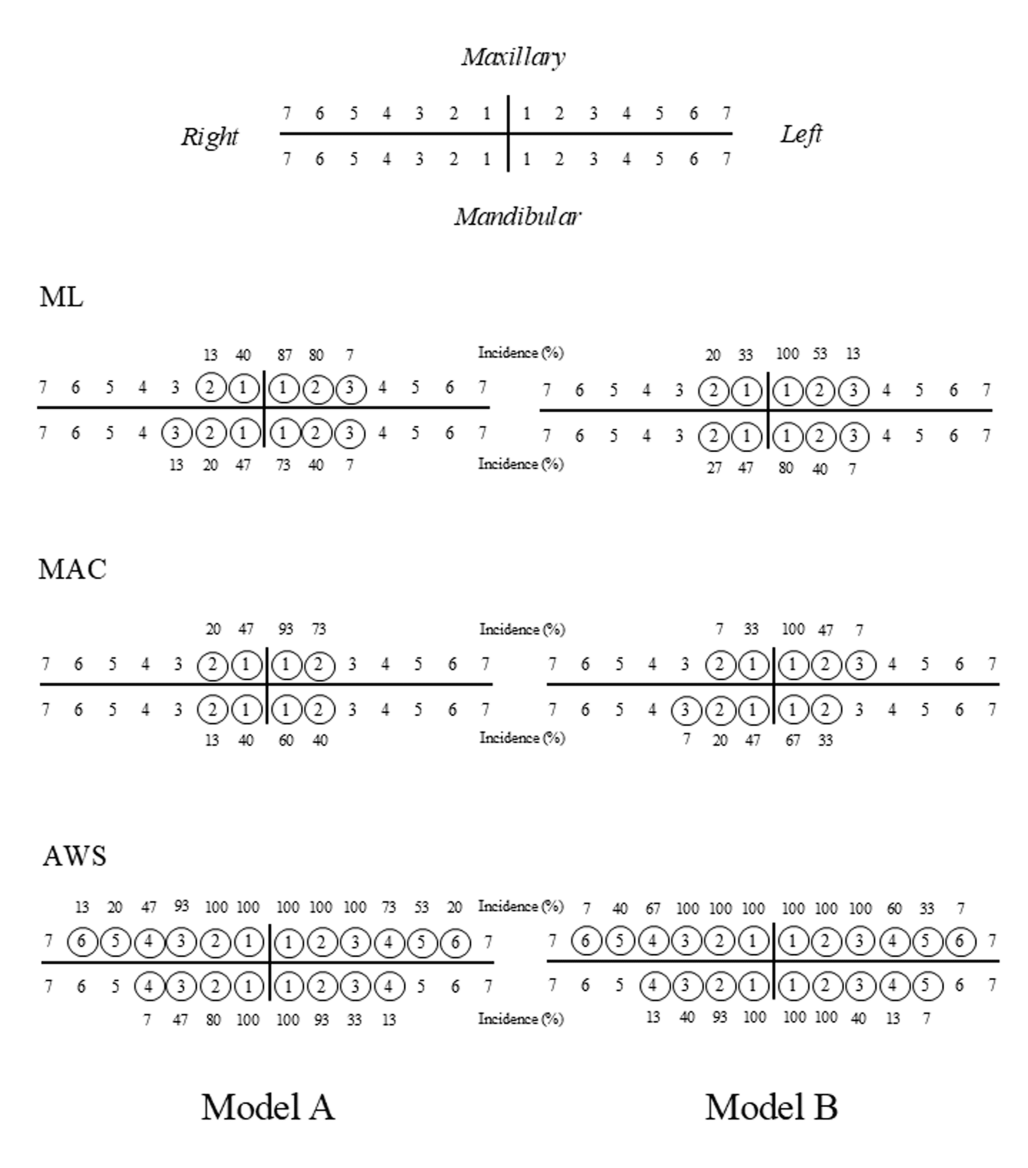
Incidences of contact between the teeth and the laryngoscope blades The contacted teeth are shown as numbers with a circle. Tooth number: 1: central incisor; 2: lateral incisor; 3: canine; 4: first premolar; 5: second premolar; 6: first molar; 7: second molar ML: Macintosh laryngoscope; MAC: McGrath MAC® video laryngoscope, AWS: Pentax-AWS Airway Scope®

The incidences of contact of individual teeth in each of the models are shown in Figure 3. In both models, higher contact rates were seen for the left maxillary central incisor (>80%) and left mandibular central incisor (>60%), with no contact with the molars when using both the ML and MAC. When using the AWS, higher contact rates were seen with the maxillary (>90%) and mandibular incisors (>40%) bilaterally, with the maxillary central and lateral incisors bilaterally and bilateral mandibular central incisors contacting the blade in every trial (100%). Additionally, occasional contact with the maxillary premolars bilaterally was observed (>20%).

## Discussion

The present simulation study showed that the teeth that most commonly come in contact with the laryngoscope blade during orotracheal intubation are the maxillary incisors, irrespective of the type of laryngoscope and simulation model used. These results suggest that the maxillary incisors are prone to injury when using any of the laryngoscopes. Additionally, this study showed that dental sites other than the anterior maxillary teeth sometimes come in contact with the laryngoscope blade during orotracheal intubation, suggesting that dental injury due to contact with the laryngoscope blade can occur at sites other than the anterior maxilla as well. This study was conducted using two kinds of simulators to avoid biased results based on the characteristics of the simulator, although both simulators are specialized for training in airway management, and their oral tissues, including teeth, accurately simulate human tissues. Therefore, the present results, indicating minimal differences between the models in the number of contacted teeth, the types of teeth contacted, and their contact rate, would be beneficial in clinical practice.

In this study, when using the ML and MAC laryngoscopes, their blades contacted both the mandibular and maxillary incisors with a high frequency. Additionally, the contact rates of both the maxillary and mandibular incisors were higher on the left than on the right side. Considering that the shapes of these blades are similar, with a greater blade height on the left side, the result that both the left-sided maxillary and mandibular incisors are at greater risk of injury, with similar incidences when using the ML and MAC, seems reasonable. However, although the fact that maxillary incisors, especially those on the left side, are the most common site of injury is already known, mandibular incisors are rarely injured in clinical practice [5,6]. In a previous comparison between using the ML and MAC, there was no significant difference in the frequency of dental injuries between them [10]. However, some reports have suggested that dental injury tends to occur more often when using the ML, since the contact force between the maxillary incisors and laryngoscope blades is lower when using the MAC than when using the ML [7,8]. Based on the above, although the present study showed similar numbers of contacted teeth and contact rates of the incisors when using the ML and MAC, the contact force might have been different between the maxillary and mandibular incisors and between these laryngoscopes, respectively. Distinct from the similar results with the ML and MAC, the number of contacted teeth per trial was higher, and the blade sometimes contacted the molars, especially the maxillary molars, when using the AWS in this study. These results were considered to reflect the fact that the blade of the AWS is thicker and wider than the other laryngoscope blades and its manipulation requires pressing on the hard palate to visualize the larynx. Considering the results of the contact status in this study, multiple parts of the dental arch might be at greater risk of injury when using the AWS. However, there is no evidence of a greater incidence of dental injury when using the AWS [11], except for a study in children that reported more frequent dental injuries when using the AWS than the ML [12]. Similar to the results when using the MAC in previous studies, some simulation studies have demonstrated that both tooth contact forces and the rate of teeth clicks were lower when using the AWS as compared to the ML [13,14]. This suggests that the net compressive force on each contacted tooth when using the AWS might be small, since the blade contacts many of the teeth during tracheal intubation, resulting in the distribution of the applied force.

As stated above, although our simulation study could identify the teeth that are likely to come in contact with each of the laryngoscope blades during tracheal intubation, the results might not necessarily correspond to clinical situations. Teeth that are in poor condition, such as due to dental caries, restored teeth, and mobile teeth, are more prone to damage, and preoperative oral examination should be performed to minimize the risk of dental injury during tracheal intubation [15-17]. Therefore, the results of this study are considered to be useful as a reference for determining which teeth should be prioritized for examination and treatment in preoperative oral examination aimed at preventing dental injuries. The research findings may also aid in selecting a laryngoscope less likely to cause dental injury for each patient based on preoperative oral examination results.

There are several limitations to the present study. First, this was only a simulation study. Since the sizes and forms of the teeth, dental arch, and jawbone, as well as the degree of mouth opening, etc., vary widely in individual patients, the tooth contact status during tracheal intubation might not always correspond with the study results. Second, the tooth contact was evaluated only in the sniffing position in this study. The sniffing position may not always be optimal depending on the type of laryngoscope used, so in clinical practice, tooth contact may vary depending on the position of the patient's head. Third, transfer of color to the teeth due to contact with the laryngoscope blade could have occurred during any of the series of maneuvers involved in tracheal intubation, including insertion and extraction of the laryngoscope blade. Thus, the teeth did not necessarily come in contact with the blade at the time of laryngeal visualization. Fourth, this study only tested the use of a regular-sized blade, although there are different blade sizes for each of the laryngoscopes used in this study. Additionally, the size and form of the ML blades are slightly different from those of the other laryngoscopes. Therefore, the tooth contact status when using other sizes and forms of each of the laryngoscope blades might differ from those observed in this study. Finally, contact force with the laryngoscope is an important factor in the occurrence of dental injuries, but this point was not evaluated in this study.

## Conclusions

This simulation study identified which individual teeth would be frequently contacted for blades of various laryngoscopes, including ML, MAC, and AWS, during orotracheal intubation. The results suggest that both the mandibular incisors and maxillary incisors are frequently contacted by laryngoscope blades when using any of these devices. In addition, when using the AWS, more teeth come into contact with the blade compared to other laryngoscopes, and there is also the possibility of contact with the molars. Understanding which teeth are more likely to come into contact with the laryngoscope blade, as suggested by this study's findings, can help anesthesiologists reduce dental injuries and guide preoperative assessments.
